# Modulating Cortical Instrument Representations During Auditory Stream Segregation and Integration With Polyphonic Music

**DOI:** 10.3389/fnins.2021.635937

**Published:** 2021-09-24

**Authors:** Lars Hausfeld, Niels R. Disbergen, Giancarlo Valente, Robert J. Zatorre, Elia Formisano

**Affiliations:** ^1^Department of Cognitive Neuroscience, Maastricht University, Maastricht, Netherlands; ^2^Maastricht Brain Imaging Centre (MBIC), Maastricht University, Maastricht, Netherlands; ^3^Cognitive Neuroscience Unit, Montreal Neurological Institute, McGill University, Montreal, QC, Canada; ^4^International Laboratory for Brain, Music and Sound Research (BRAMS), Montreal, QC, Canada; ^5^Maastricht Centre for Systems Biology (MaCSBio), Maastricht University, Maastricht, Netherlands; ^6^Brightlands Institute for Smart Society (BISS), Maastricht University, Maastricht, Netherlands

**Keywords:** polyphonic music, auditory scene analysis, auditory stream segregation, envelope tracking, EEG, attention

## Abstract

Numerous neuroimaging studies demonstrated that the auditory cortex tracks ongoing speech and that, in multi-speaker environments, tracking of the attended speaker is enhanced compared to the other irrelevant speakers. In contrast to speech, multi-instrument music can be appreciated by attending not only on its individual entities (i.e., segregation) but also on multiple instruments simultaneously (i.e., integration). We investigated the neural correlates of these two modes of music listening using electroencephalography (EEG) and sound envelope tracking. To this end, we presented uniquely composed music pieces played by two instruments, a bassoon and a cello, in combination with a previously validated music auditory scene analysis behavioral paradigm (Disbergen et al., 2018). Similar to results obtained through selective listening tasks for speech, relevant instruments could be reconstructed better than irrelevant ones during the segregation task. A delay-specific analysis showed higher reconstruction for the relevant instrument during a middle-latency window for both the bassoon and cello and during a late window for the bassoon. During the integration task, we did not observe significant attentional modulation when reconstructing the overall music envelope. Subsequent analyses indicated that this null result might be due to the heterogeneous strategies listeners employ during the integration task. Overall, our results suggest that subsequent to a common processing stage, top-down modulations consistently enhance the relevant instrument’s representation during an instrument segregation task, whereas such an enhancement is not observed during an instrument integration task. These findings extend previous results from speech tracking to the tracking of multi-instrument music and, furthermore, inform current theories on polyphonic music perception.

## Introduction

Listening to a sound of interest in an environment with multiple competing sounds represents a common though challenging task that the auditory system solves seemingly without effort. When this sound of interest is music, our auditory system segregates it further into its individual components (or streams) which represent, for example, multiple simultaneously playing instruments. The perceptual mechanisms for analyzing and resolving auditory (and musical) scenes have been described in a comprehensive theoretical framework by Bregman (1990). Research inspired by Bregman’s theory has detailed the conditions under which acoustical scene elements are segregated or integrated, the processes of which are driven by physical differences between sounds (i.e., bottom-up) as well as by top-down mechanisms, among which is the listener’s focus of attention (Bregman, 1990; Brochard et al., 1999; Cusack et al., 2004; Carlyon and Cusack, 2005; Besle et al., 2011; Lakatos et al., 2013; Riecke et al., 2016). Here, we focus on the contributions of top-down attentive processes to auditory scene analysis (ASA) in the context of multi-instrument music listening (e.g., McAdams and Bregman, 1979; Bregman, 1990; Bey and McAdams, 2003).

Most studies investigating ASA mechanisms have employed simple auditory scenes such as pure tones in noise or alternating tone sequences (Bregman, 1990, 2015; Ciocca, 2008; Alain and Bernstein, 2015). Since the auditory system has been optimized to process sounds that are relevant for behavior, naturalistic auditory scenes with ecologically valid stimuli are valuable to gain a better understanding of ASA (for a review see, Theunissen and Elie, 2014). To date, most research on ASA with naturalistic stimuli has focused on language and employed multi-speaker environments in combination with selective attention tasks. Several studies used these paradigms in conjunction with magnetoencephalography (MEG), electroencephalography (EEG), or electrocorticography (ECoG) and identified effects of selective attention using sound envelope reconstruction methods (referred to as tracking; e.g., Nourski et al., 2009; Kerlin et al., 2010; Ding and Simon, 2012b; Kubanek et al., 2013; Crosse et al., 2015; Dijkstra et al., 2015; O’Sullivan et al., 2015). This research showed that – for scenes containing two simultaneous speakers – attended speech could be better reconstructed as compared to unattended speech (Ding and Simon, 2012a,b; Mirkovic et al., 2015) at delays of approximately 100 ms or more (Power et al., 2012; O’Sullivan et al., 2015; Hausfeld et al., 2018). These results suggest an attention-mediated biasing mechanism, which enhances the neural representation of the relevant speech stream, following an initial acoustically driven analysis of the sound mixture.

This investigation of multi-speaker scenes has provided insights into the processing of speech. A generalization of these mechanisms to auditory scenes including sounds other than speech, however, is not straight-forward and requires further investigations (e.g., Alho et al., 2014). We argue that music, especially when containing multiple instruments (i.e., polyphonic), is very well suited for the investigation of ASA in naturalistic and complex listening scenarios. This type of music contains rich but acoustically well-controlled sound mixtures with a continuously varying degree of spectral and temporal overlap. Furthermore, multi-instrument music allows for the study of both the typical segregation aspect of ASA and the less investigated integration condition.

Using EEG, previous research demonstrated, in musically experienced participants, that there is a high correlation between the evoked response potentials (ERPs) and the envelopes of 3-s musical stimuli, peaking at 100 ms after sound-onset (Schaefer et al., 2011). They proposed that these correlations are mostly representative of bottom-up processing and potentially occur outside the focus of attention. Treder et al. (2014) reported similar effects, even though during later delays around 200 ms post-stimulus onset. They compared ERP responses for attended and unattended instruments within multi-instrument music that contained standard or deviant structures within the individual instruments. Their results suggest that higher-level cortical processing influenced the ongoing sound representations, specifically of the to-be-attended instrument. Taken together, these studies indicate that music envelopes are represented in the EEG signal and are, similarly to speech, modulated by attention during middle to late time-windows. These studies should be interpreted with caution since investigations of music stream representation and attentive modulation have mostly focused on expert musicians, who typically display modified listening behavior as compared to non-musicians (Coffey et al., 2017; e.g., Puschmann et al., 2018). Very few studies have investigated the processes involved in auditory stream integration, and even less have used music stimuli (Sussman, 2005; Uhlig et al., 2013; Ragert et al., 2014; Disbergen et al., 2018).

A functional magnetic resonances imaging (fMRI) study employing a music ASA paradigm (Disbergen, 2020, chapter 3) demonstrated that segregating or integrating music instrument resulted in differential cortical activity patterns in a large frontal-temporal network of sound-responding cortical regions. This network included several regions early in the auditory processing hierarchy, such as Heschl’s gyrus (HG). Even though fMRI is well suited to localize the effects of attention, it is less well able to determine the time-course and order of effects. For example, results in HG could have originated from both an early modulation of the initial bottom-up driven sound analysis as well as later top-down driven mechanisms that influence sustained responses in HG through feedback connections. In this study, we investigated these attention effects with a high temporal resolution to identify the temporal development of these attentive effects. To this end, we employed the previously validated psychophysical paradigm (Disbergen et al., 2018) in combination with an EEG-based envelope-based neural tracking method (O’Sullivan et al., 2015; Crosse et al., 2016). Non-musicians performed listening tasks which required them to segregate or integrate auditory streams formed by custom-composed polyphonic music pieces, attending either a single instrument or integrating across both melodies, respectively.

During the segregation condition, we expected higher tracking accuracy when an instrument was attended to as opposed to unattended. Effects were predicted at delays beyond 100 ms due to earlier time-windows mostly representing initial bottom-up mechanisms, driving the stimulus processing based on acoustical features. In general, early windows are not expected to be strongly biased by attentive mechanism, albeit modulations may already take place (Poghosyan and Ioannides, 2008). Integration of instruments was hypothesized to differ from segregation mostly regarding its timing, since integration can typically be understood as a cognitively higher-level task as compared to segregation, hence potential differences are expected to emerge during later delay-windows for instrument integration as compared to segregation.

## Materials and Methods

### Participants

Nineteen adult volunteers (10 women; age 23.9 ± 3.3 years, mean ± standard deviation) with self-reported normal motor and vision abilities participated in this study. All participants displayed normal hearing thresholds (<25 decibels Hearing Level), as measured by pure-tone audiometry in both ears at frequencies of 0.25, 0.5, 0.75, 1.0, 2.0, 3.0, 4.0, and 6.0 kHz. None of the participants spoke a tonal language and all had less than 2 years of (formal) musical training on a lifetime basis with instruments which were not included in this study, i.e., bassoon or cello, as assessed via the Montreal Music History Questionnaire (Coffey et al., 2011). Volunteers were students from Maastricht University who provided written informed consent prior to the experiment in accordance with the protocol as approved by the Maastricht University Ethics Review Committee Psychology and Neuroscience (#167_09_05_2016). Four participants were excluded from the EEG analysis due to low behavioral performance metrics in one or multiple conditions, hence subsequent analyses were performed on 15 participants (Supplementary Table 1). More specifically, accuracy of task performance were inspected as well as the false alarm (FA) rates in “no-target” and “opposite voice target” trials (see “Stimuli” and “Results” sections) to better ensure no response bias between these trial categories. Such response biases (high FA rates for “opposite voice targets” in combination with low FA rates for trials without targets or vice versa) potentially indicate strategies not relying on instrument segregation and paying attention to the relevant instrument but attention to temporal features in either instrument (see Supplementary Table 1). Participants with accuracy values lower than the lower quartile − interquartile range (i.e., Q1-IQR) and differences in FA rates higher than the upper quartile + interquartile range (i.e., Q3+IQR) were considered as outliers. None of the participants took part in the previous studies using the same paradigm and stimuli (i.e., Disbergen et al., 2018; Disbergen, 2020, chapter 3).

### Stimuli

In this experiment, we employed a previously validated psychophysical paradigm for the study of ASA with multi-instrument music (Disbergen et al., 2018). An in-depth discussion of task and training as well as a demonstration of the paradigm’s validity also in non-musically trained participants can be found in Disbergen et al. (2018). Twenty custom-composed polyphonic counterpoint music pieces (28 s duration) consisting of two instrument voices were synthesized for bassoon (treble clef) and cello (bass clef) at a tempo of 60 beats per minute. Melodies were synthesized independently for bassoon and cello from musical instrument digital interface (MIDI) files, with a sampling rate of 44.1 kHz and a 16 Bits resolution in Logic Pro 9 (Apple Inc., Cupertino, CA, United States). Resulting stimuli were combined *post-hoc* into polyphonic pieces with root mean square (RMS) equalization across the full length of compositions for each instrument (i.e., each instrument had the same RMS in each composition) and their onsets and offsets exponentially ramped with a rise-fall time of 100 ms. All stimulus processing and manipulation aside from synthesizing was performed with custom-developed MATLAB codes (The MathWorks Inc., Natick, MA, United States).

**FIGURE 1:**
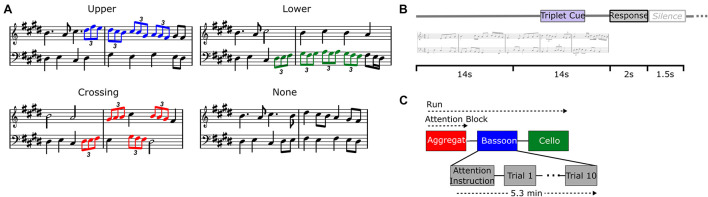
Experiment design. Different triplet versions for each music composition **(A)**: upper voice (i.e., bassoon; blue notes), lower voice (i.e., cello; green notes), crossing voices (red notes), no triplets. Trial buildup **(B)** with 28 s stimulus, 2 s response window, and 1.5 s silence. Trials were presented in attentive blocks of 10 stimuli each and preceded by a visual attention instruction and silence **(C)**.

We examined the neural modulations of musical instrument tracking both during the integration versus segregation conditions as well as within the segregation condition where we compared attended versus unattended tracking. To achieve these different listening contexts within a fixed acoustic setting, we varied the listener’s focus of attention using a temporal detection task which was implemented through rhythmic modulations that were incorporated within the polyphonic music (see Disbergen et al., 2018). Rhythmic modulations in the music comprised four consecutive triplets with a total duration of 4 s, each containing three eighth notes played in one single beat and carefully integrated into the melodic structure (Figure 1). Patterns of four consecutive triplets were located in the upper voice melody (i.e., bassoon; Figure 1A, blue notes), lower voice (i.e., cello; Figure 1A, green notes), across voices (Figure 1A, red notes), or not present.

If triplets were located across instruments, they started randomly in bassoon or cello and alternated voices accordingly, while patterns present in a single voice were only located within that respective instrument. Triplets were always incorporated in the second half of the melodies, pseudo-randomly starting between 14 and 19 s after music onset, resulting in stimuli which were physically identical up until triplet occurrence. Rhythmic (i.e., temporal) modulations in the form of triplets were chosen due to their orthogonality toward pitch-based segregation mechanisms, facilitating their detection by listeners with little to no musical training.

### Paradigm

Due to the limited musical education of participants, they were first subjected to a separate *training session* which took place between one to 5 days before the main experiment. During the training session, participants listened to music of slowly increasing complexity; initiating with scales including individual triplets and completing with melodies containing triplet patterns at equal complexity as the actual experiment. During the final training blocks a performance of 85% accuracy was required to proceed to the next block with more complex stimuli. The training session concluded with a pre-test including 24 trials similar to the ones in the main experiment (i.e., eight trials for each attention task) and an accuracy of 85% was required for participants to enter the main experiment; for training details see Disbergen et al. (2018).

During the *test session* including EEG acquisition, listeners were instructed to complete a forced-choice delayed-response target detection task within or across instruments, attending the same instrument(s) during an attention block of 10 consecutive trials (Figure 1C). Each trial comprised the music stimulus of 28 s, a 2-s response window, and a 1.5-s silence (Figure 1B). A visual instruction was presented before the beginning of each attention block, cuing which instrument(s) to attend: bassoon, cello, or aggregate (i.e., both instruments; Figure 1C). After the stimulus ended, listeners responded via a button-press whether the triplet pattern was present in those instrument(s) they were instructed to attend. Stimuli were presented pseudo-randomly in sets of three consecutive attention blocks of 10 trials each, covering all three attention conditions. This three-block scheme was repeated four times, covering all stimuli under all attention conditions twice, hence resulting in two fully balanced experiment repetitions. Each attention block of 10 trials contained 5 target trials and 5 non-target trials in random order. For the bassoon and cello tasks, blocks included five target trials with triplets in the task-relevant instrument (i.e., in the upper or lower voice during the bassoon or cello task, respectively) and five non-target trials. Of these five non-target trials two or three trials contained triplets in the task-irrelevant instrument (i.e., in the lower or upper voice for the bassoon or cello task, respectively) and two or three trials did not contain triplets. The number of these two types of non-target trials per block was pseudo-randomized across blocks and alternated between experiment repetitions. For the aggregate task, blocks included five trials with triplets crossing voices (i.e., target) and five trials without triplets (see also Disbergen, 2020).

### EEG Data Acquisition and Pre-processing

Electroencephalographic data was recorded in an electrical insulated and sound attenuated chamber from 63 electrodes using BrainAmp amplifiers (Brain Products, Munich, Germany) in a modified 10–20% electrode system (EasyCap, montage 11) and referenced to electrode TP9. The vertical and horizontal electrooculograms (EOG) were recorded from electrodes placed below and next to the right eye. During acquisition, the electrodes’ impedance was kept below 5 k. The EEG signal was bandpass filtered with an analog filter at cutoffs 0.01 and 200 Hz and digitized at a 500 Hz sampling rate. EEG data pre-processing was performed using the EEGLAB toolbox (Delorme and Makeig, 2004) in MATLAB and custom MATLAB codes. Pre-processing steps included band-pass filtering with a finite impulse response (FIR) filter at cutoffs 0.5 and 45 Hz, re-referencing to an average electrode reference, and epoching from 1 to 28 s relative to the onset of the auditory stimulus. An independent component analysis (ICA), as implemented in the EEGLAB *runica.m* function, was used on the epoched data for artifact removal. This component estimation was followed by a manual definition of artifact components containing eye movements, blinks, muscle activity, and channel noise. EOG and component statistics were employed to aid artifact identification in addition to visual inspection of component time courses, weight topographies and spectra. For each participant, artifact components were removed (4.7 ± 1.9, group mean ± standard deviation) and data from remaining components was back-projected into sensor space. Finally, the pre-processed EEG data was re-epoched from 2 to 14 s to exclude activity related to both initial streaming processes and motor responses as well as any possible modulations caused by the presence of triplets in the second half of the stimulus.

### Analysis

#### Behavioral Analysis and Sound Envelope Estimation

**FIGURE 2:**
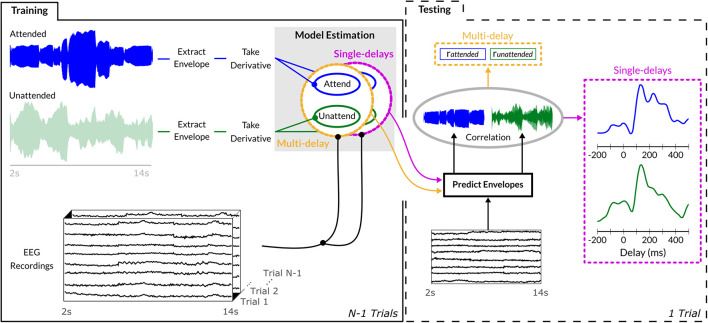
Sound envelope tracking method. Envelopes were extracted from each instrument’s waveform via an absolute Hilbert transform, its derivative was employed to estimate both single-delay and multi-delay envelope models on *N*-1 training trials. To assess generalization, the estimated envelope model was used to predict the sound envelope of the single unseen trial and its output correlated with the trial’s actual sound envelope. Multi-delay models provided output in a single correlation value encompassing the evidence of all delays between 0 and 400 ms, while the single-delay models generated a correlation value for each individual delay between –200 and 500 ms (10-ms step size).

Behavioral responses were classified as hits, misses, false alarms, and correct rejections per condition, and, due to possibly differing number of trials across participants, reported as percent accuracy. Sound onset envelopes were extracted from the music stimuli and used in combination with EEG data to train a sound-envelope model *E* (i.e., decoder) separately for bassoon (*E_b_*) and cello (*E_c_*; Figure 2). Sound envelopes were extracted by determining the absolute Hilbert transform of each instrument independently and passing the resulting signal through a low-pass filter with a cutoff of 8 Hz, of which the derivative was taken and half-wave rectified; see Hausfeld et al. (2018) for a similar approach. Such processing emphasizes short-term sound intensity fluctuations, salient in both the ongoing low-frequency EEG signals as well as in music (Sturm et al., 2015; Fiedler et al., 2017; Petersen et al., 2017); for brevity, we will refer to sound onset envelopes as sound envelopes unless further specification is required.

### Sound Envelope Modeling

Similar to previous EEG studies investigating envelope tracking (Mirkovic et al., 2015; O’Sullivan et al., 2015; Fuglsang et al., 2017), we adopted a deconvolution approach which fits, for each trial *k*, a multi-delay model *g* using the sound envelope *E_k_* and EEG data *X_k_* from 63 channels across 41 delays between 0 and 400 ms (i.e., 10 ms step-size). Analyses were performed in Matlab (The MathWorks Inc., Natick, MA, United States) using the mTRF toolbox (Crosse et al., 2016) and custom-made scripts. The convolution kernel *g_k_* was estimated by L2-regularized least-squares regression:


$$g_{k}={\left(X_{k}^{T}X_{k}+\lambda I\right)}^{-1}X_{k}^{T}E_{k}.$$

Regularization was performed using the identity matrix *I*, with the regularization parameter set to λ = 10^4^ for both tasks and for all participants; this choice was based on a previous study by Hausfeld et al. (2018) using the same EEG setup. The EEG data matrix *X_k_* was constructed by concatenating the responses of all EEG channels and delays for the presented sound envelope at each individual time-point *t*, resulting in *g_k_* with dimension 1201 (time points) × 2583 (channels × delays). Independent test data and sounds were employed to evaluate models on their generalization capacity to reconstruct/predict the onset envelopes from unseen bassoon, cello, or aggregate tracks ($\hat{E_{b}}$, $\hat{E_{c}}$, and $\hat{E_{a}}$, respectively). Model prediction and matches to sound envelopes from the test data sets were assessed with Pearson’s correlation coefficient *r* (Ding and Simon, 2012a; O’Sullivan et al., 2015). Generalization performance was tested within a leave-one-trial-out scheme, averaging the *N*-1 decoders of the training trials and applying this to the EEG data of the remaining test trial; this procedure was repeated for all trials and the correlations were averaged. The decoder *g_i_* applied to test trial *i* was estimated as


$$g_{i}=\frac{1}{N-1}\sum_{j\neq k}g_{j}$$

reconstructing the unseen trial’s envelope $\hat{E_{i}}$ by convolution


$$\hat{E_{i}}=g_{i}X_{i}^{T}.$$

### Envelope Model Estimation and Statistical Comparison

Within the segregation conditions, we computed models for bassoon and cello independently across all respective trials. This resulted in four different decoders: bassoon in the bassoon task ($\hat{E_{b}^{b}}$), cello in the bassoon task ($\hat{E_{c}^{b}}$), and *vice versa* ($\hat{E_{b}^{c}}$ and $\hat{E_{c}^{c}}$, respectively). For the aggregate ($\hat{E_{a}}$), we estimated the decoder based on the envelope of the waveform derived from adding the waveforms of the two instruments for each of the three tasks (i.e., bassoon, cello, and aggregate task).

Statistical comparisons of task or decoder differences for the *multi-delay* models were performed by non-parametric Wilcoxon signed-rank tests. Effect sizes for this test were defined as $r_{\text{eff}}=\left|z\right|/\sqrt{N}$, where *z* is the test statistic (normal approximation) of the sign-rank test; values of 0.5, 0.3, and 0.1 are considered as large, medium and small effects, respectively (Fritz et al., 2012). In order to gain further insight into those EEG delays which contribute to envelope decoding, we adopted an identical approach as above, only restricting training and testing to single delays as opposed to multiple ones. This *single-delay* approach similarly employed *X_k_*, the measurements of all channels for all time points although only at a single delay. In comparison to the analysis with the multi-delay model that provides a single value indicating overall tracking performance, an analysis with many single-delay models results in a *tracking profile* which indicates the tracking performance for each individual delay. In total 71 single delays were tested between −200 and 500 ms; differences between models were assessed by employing a Wilcoxon signed-rank test and subsequent multiple comparison correction by a cluster-size based permutation test (Maris and Oostenveld, 2007). More specifically, we tested for each delay whether two conditions differed significantly using Wilcoxon’s sign-rank test (*p* < 0.05) and then summed the corresponding *z*-values of consecutively significant delays to obtain for each cluster its *z*_sum_. These values were then compared to an empirical null distribution obtained by permuting labels of conditions for each participant (*n*_perm_ = 2^14^). For each permutation, clusters of significant differences were determined and the maximal *z*_sum_ values were extracted. This process was repeated for all permutations, each contributing a single measure to the distribution of *z*_sum_ values under chance given the data. Comparison of true-label values with this distribution, resulted in a probability estimate corrected for multiple comparison, and those clusters which passed the *p* < 0.05 threshold were labeled as significant. This cluster-based multiple comparison correction was done separately for each tracking profile.

Empirical chance level performance of the decoding models was estimated by performing the analysis as discussed, albeit with phase-scrambled versions of the stimuli (*n*_scramble_ = 10^4^). Such an approach keeps the frequency components of the envelopes constant. Average model performance obtained from these scrambled envelopes was compared to the non-scrambled tracking performance. Note that if instrument envelopes were to be permuted, chance level would be overestimated due to the preservation of temporal note onsets between trials (cf. “Stimulus” section, Disbergen et al., 2018).

In order to gain further insight into active mechanisms during the integrative condition, we fitted the aggregate single-delay tracking profile (*r*_agg_) for each participant from a linear combination of the individual instrument tracking profiles obtained during the aggregate task ($r_{b}^{a}$ and $r_{c}^{a}$) using ordinary least-squares estimation:


$$r_{agg}=\beta_{0}+\beta_{b}r_{b}^{a}+\beta_{c}r_{c}^{a}+\varepsilon$$

where β_*b*_, and β_*c*_ are coefficients of the instrument time courses, β_0_ a constant and ε∼ *N*(0, σ^2^) the error term.

#### Channel Contributions

To further disentangle which EEG channels potentially contributed to the segregation condition’s tracking performance, we adopted a leave-one-channel-out approach for the single-delay models. The tracking of sound envelopes was achieved identically as above, only leaving one channel out for each iteration. Single-delay decoders for trial *k* were trained on data $X_{k}^{c}$ (1201 [time points] × 2542 [62 channels × 41 delays]), where *c* denotes the index of the left-out channel. Tracking correlations of the leave-one-channel-out datasets were subtracted from the performance achieved with the full dataset and visualized as scalp topographies. A lower tracking performance of the left-out model, i.e., negative values in the topographies, indicates that the respective channel possesses information relevant for the model’s observed sound envelope tracking.

### EEG Prediction Analysis

To take co-variation of instrument envelopes into account, we performed a cross-validated encoding analysis (i.e., EEG prediction) with models differing in their complexity including single, pairs or all envelope predictors of the bassoon and cello instruments to model the single channel EEG signal. Similar to the reconstruction by multi-delay models, we provide an interval of potential lags between 0 and 400 ms and perform the encoding analysis with the mTRF toolbox (Crosse et al., 2016). Encoding models are trained in a leave-trial out manner and their performances are tested with the unseen trial similar to the tracking/decoding analysis. Given the model’s complexity and following the previous analysis, models were trained with regularized least-squares regression using grid search to optimize the regularization parameter λ = 10^x^ where *x* = {−5, −4, …, 0, 1, …, 5}.

## Results

**FIGURE 3:**
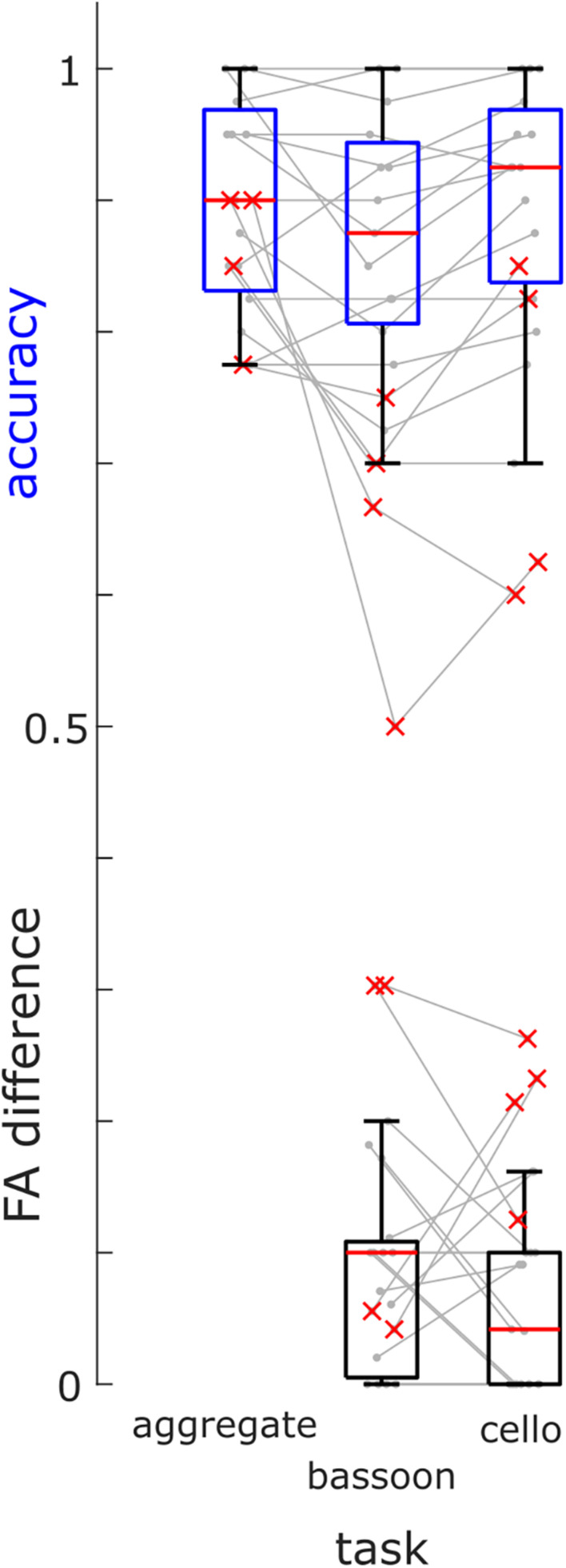
Group Behavioral Results. Accuracies of triplet detection across all tasks (blue boxes) and FA differences between trials with triplets in the other instrument and trials without triplets (black boxes) for the bassoon and cello task for all participants (box = 25th percentile–median–75th percentile). Gray lines denote performances of single participants, red crosses indicate participants excluded from further analysis.

Based on their low accuracy (i.e., [hits + correct rejections]/#trials) and high differences in FA rates during selection tasks, we removed four participants (Figure 3, red crosses) from further analysis (note that re-analyzing EEG data did not change findings qualitatively). Overall, participants completed the experiment at high accuracy for all attention tasks: bassoon (0.875 [0.138], median [interquartile range]), cello (0.925 [0.131]) and aggregate (0.900 [0.138]; Supplementary Table 1). We observed differences between tasks for both accuracy (χ^2^(2) = 6.83, *p* = 0.033; Friedman test) and FA rates (χ^2^(2) = 10.86, *p* = 0.004). *Post-hoc* tests indicated lower accuracy for triplet detection during the bassoon task versus the cello task (*z* = −2.701, *p*_FDR_ = 0.021; Wilcoxon sign-rank test, multiple comparison corrected using false discovery rate (Benjamini and Hochberg, 1995) as well as lower false alarms rates for the integration versus segregation tasks (aggregate versus bassoon: *z* = −2.841, *p* = 0.014; aggregate versus cello: *z* = −2.252, *p* = 0.037). The higher FA rate could be due to triplets in the task-irrelevant instrument for segregation tasks. The participant’s criterion *C* (computed after loglinear transformation) did not differ between tasks (χ^2^(2) = 4.96, *p* = 0.084, Friedman test) indicating a similar response behavior across tasks. Furthermore, for segregation tasks, no difference was observed in FA rates during trials that contained triplets in the unattended instrument as compared to trials without triplets (*z* = 1.366, *p* = 0.172, Supplementary Table 1).

### Sound Envelope Tracking of Music

**FIGURE 4:**
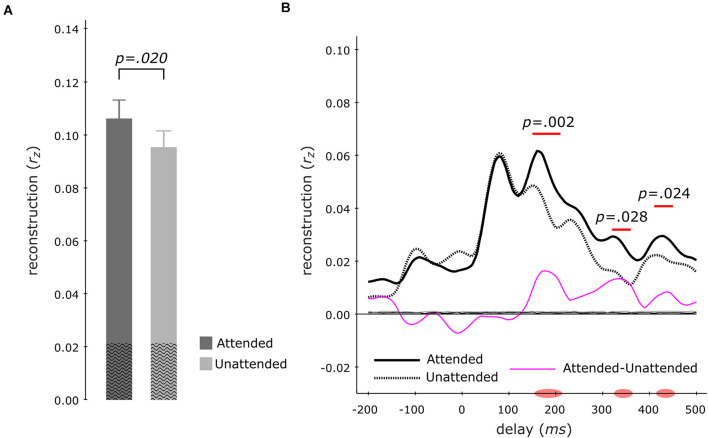
Envelope Tracking during the Segregation Tasks. Multi-delay model tracking performance **(A)** for attended (dark gray) and unattended (light gray) instruments, showing a significant difference between the two listening conditions. The average empirical chance-level is displayed as superimposed black waves. Single-delay tracking profiles **(B)** showing a significant delay-resolved difference between attended (thick black solid line) or unattended (thick black dashed line) instruments during 150–220 ms, 320–360 ms, and 410–450 ms. The thin purple line shows the difference between attended and unattended tracking; the thin solid black (attended) and thin dashed black (unattended) lines present the average empirical chance-level.

To examine the neural representation of the attended instrument in the segregation conditions, we analyzed the data pooled across both instruments when attended versus unattended. Correlating the envelope predictions from the multi-delay models with actual envelopes of test-trials revealed that–during segregation trials–the attended instruments (*r*_*z*_ = 0.107 ± 0.007; mean ± s.e.m) displayed significantly better tracking versus unattended instruments (*r*_*z*_ = 0.096 ± 0.006; *z* = 2.329, *p* = 0.020, *$\bar{x}$*_att–unatt_ = 0.011, *r*_eff_ = 0.60; Wilcoxon signed-rank test; Figure 4A). Analysis of the same data with single-delay models indicated significantly higher tracking for the attended instruments at 150–210 ms (*p* = 0.002) and at late 320–360 ms (*p* = 0.028) as well as 410–450 ms (*p* = 0.024) delay windows (cluster-size based permutation test; Figure 4B).

**FIGURE 5:**
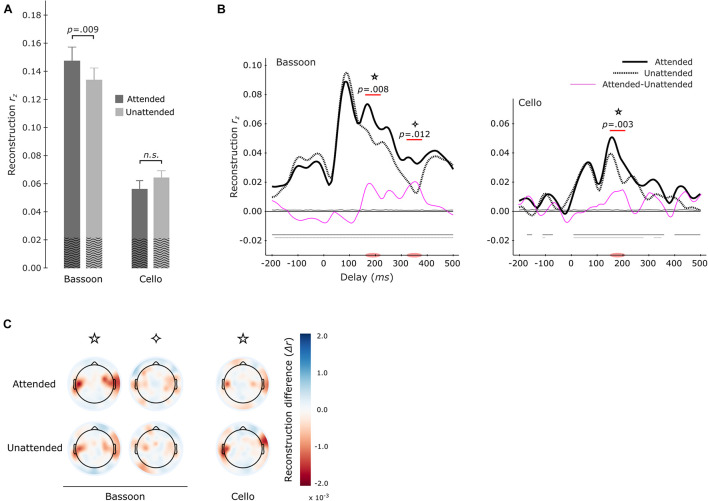
Envelope tracking during the segregation tasks per instrument. Multi-delay model tracking performance **(A)** for attended (dark gray) and unattended (light gray) instruments. The tracking of bassoon and cello envelopes is displayed at left and right two bars, respectively. Horizontal lines and values above denote results of significance testing between attended and unattended conditions for the bassoon and an interaction between attended and reconstructed instrument. The average empirical chance-level is superimposed as black waves. Single-delay tracking profiles **(B)** for the bassoon envelope (left panel) and cello envelope (right panel). Significant tracking differences between attended and unattended instruments are indicated by the purple lines. Attention effects for bassoon tracking were found during the 160–220 ms (✩) and 320–380 ms (✧) delay windows and for cello tracking only during a 150–210 ms delay window (✩). Differences between attended and unattended tracking are presented as thin pink lines. Thin horizontal lines within the plot indicate the average empirical chance-level. Horizontal lines in the negative indicate time-points which significantly differed from chance. Topographical representation **(C)** of tracking differences for the leave-one-electrode out analysis of both the attended and unattended conditions for each instrument during the significant delay-windows indicated in panel **(B)**.

Further investigations were performed into whether attended versus unattended tracking effects differed per instrument. Models were estimated separately for each instrument when attended or unattended. For example, reconstructing the envelope of the bassoon during the bassoon task versus the cello task. Overall, multi-delay tracking resulted in significantly higher envelope tracking for the bassoon compared to the cello instrument, both when instruments were attended to (*z* = 3.408, *p* < 0.001, *r*_eff_ = 0.88, *$\bar{x}$*_bassoon–cello_ = 0.084) or unattended (*z* = 3.408, *p* < 0.001, *r*_eff_ = 0.88, *$\bar{x}$*_bassoon–cello_ = 0.079). For instrument tracking with multi-delay models, significantly higher tracking was found for the bassoon during the bassoon task versus the cello task (*z* = 2.613, *p* = 0.009, *r*_eff_ = 0.68, *$\bar{x}$*_att–unatt_ = 0.014; Figure 5A, left-hand columns), while the attention effect for the cello was not significant (*z* = 1.420, *p* = 0.0156, *r*_eff_ = 0.34, *$\bar{x}$*_att–unatt_ = 0.008; Figure 5A, right-hand columns). Tracking profiles from the single-delay analysis showed that the bassoon was reconstructed better when attended during two delay windows at 160–220 ms (*p* = 0.008) and 320–380 ms (*p* = 0.012; Figure 5B, left frame). The cello displayed a higher tracking when attended at the delay window 150–210 ms (*p* = 0.003; Figure 5B, center frame), which is comparable to the first interval for the bassoon. Please note that differences in the envelopes of bassoon and cello (Supplementary Figure 1) might have affected these latencies. However, although differences exist, the decoded envelopes are rather similar between the instruments in contrast to other features related to timbre. Further understanding of the topographical contribution of EEG channels to the tracking of sound envelopes per instrument was obtained with a leave-one-channel-out approach, demonstrating that channels at temporal sites contributed most to the tracking (Figure 5C). Additionally, topographies were very similar when an instrument was attended versus unattended (Figure 5C).

**FIGURE 6:**
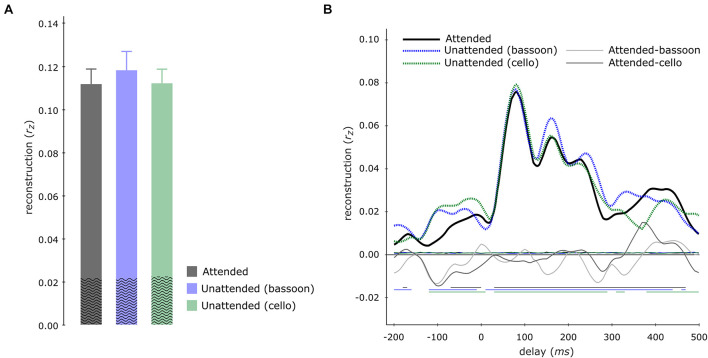
Aggregate tracking during integration and segregation tasks. Multi-delay model tracking performance **(A)** for the aggregate during the aggregate (gray), bassoon (blue), and cello (green) tasks, displaying no significant differences between model tracking capacities. The average empirical chance-level is displayed as superimposed black waves. Single-delay aggregate tracking profiles **(B)** showing tracking performance for the aggregate (black solid line), bassoon (blue dashed line), and cello (green dashed line) tasks. Differences between attention to aggregate and the attention to bassoon of tracking are shown by thin light-gray and dark-gray lines, respectively. Thin horizontal wavy lines within the plot indicate the average empirical chance-level. Horizontal lines at negative tracking values at the bottom of the graph indicate those time-points which displayed significant tracking performance.

Contrary to our hypothesis, tracking of the aggregate envelope was not significantly higher during the aggregate task compared to the segregation tasks, neither for multi-delay (aggregate versus bassoon task: *z* = −1.988, *p*_FWE_ = 0.094; aggregate versus cello task: *z* = −0.114, *p*_FWE_ = 0.910) nor single-delay models (Figure 6).

Next, we tested for each participant how the bassoon and cello envelopes contributed to the tracking of the aggregate. To this end, we fitted the individual aggregate single-delay tracking profile with a linear combination of the individual instrument tracking profiles obtained during the aggregate task (Supplementary Figure 2). Our results showed that the aggregate tracking profile was best fitted by higher coefficients of the tracking profile for the bassoon (β_bassoon_ = 0.695) in comparison to the cello instrument (β_cello_ = 0.440) across participants (*z* = 3.012, *p* = 0.003, *r*_eff_ = 0.78). This suggests that tracking profiles for the aggregate resemble more the profiles of the bassoon than the cello instrument. This might be due to the tendency of higher similarity of the aggregate envelope to the bassoon as compared to cello envelopes (*r*_bassoon/aggregate_ = 0.719 versus *r*_cello/aggregate_ = 0.648, *t*(39) = 1.93, *p* = 0.06; across 40 stimuli).

**FIGURE 7:**
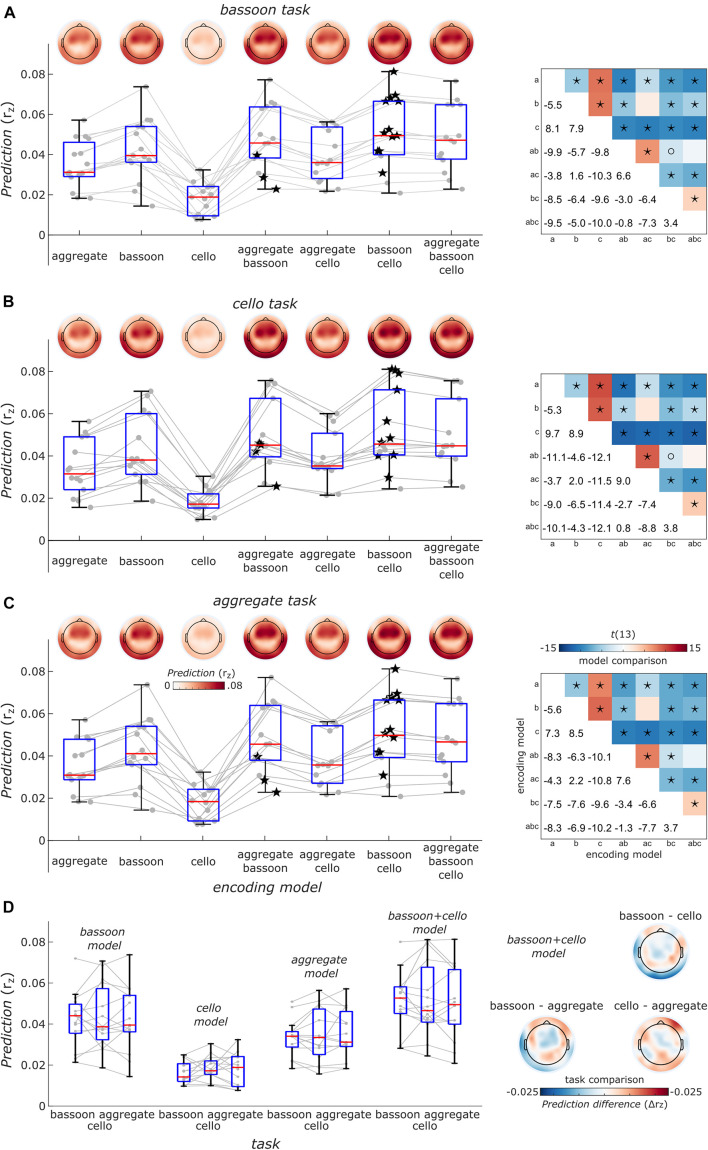
Overview of EEG prediction performance. **(A)** Encoding models prediction performance for EEG data acquired during the bassoon task. Boxplots show the average model performance of different encoding models across all 63 channels. Boxes indicate the interquartile range, red lines indicate the median and whiskers reach to the most extreme data point up to 1.5 interquartile from the lower or upper quartile. Gray lines and dots denote encoding performance for individual participants. Encoding results are presented as a function of models reflecting the envelope the bassoon, cello or the aggregate or their combination with 2 or 3 predictors. Best models for individual participants are indicated by star symbols. Topographic plots show prediction performance for single channels. Right: matrices show comparisons of different models. Asterisks and open circles indicate the significant differences for model pairs at *p*_FDR_ < 0.01 and *p*_FDR_ < 0.05, respectively (two-sided, false-discovery-rate adjusted *p*-values across 21 paired comparisons). a, b, c denote the aggregate, bassoon and cello predictors to identify different encoding models. Panels **(B,C)** same as panel **(A)** but for the cello and aggregate task, respectively. **(D)** Right: task comparisons of encoding model prediction averaged across channels indicated as in panels **(A–C)** for single-predictor models and the two-predictor model with bassoon and cello envelope. Left: prediction differences for models with the bassoon and cello predictors between tasks for single channels. Neither the average prediction across channels nor the predictions for single channels were significantly different between any pair of tasks (two-sided, uncorrected).

Finally, to take co-variation of instrument envelopes into account, we performed a cross-validated encoding analysis (i.e., EEG prediction) with models differing in their complexity including single, pairs or all envelope predictors of the bassoon and cello instruments. Notably, across tasks, the model including the bassoon and cello envelopes explained EEG data best and the full models (i.e., adding the aggregate envelope) showed lower EEG prediction performance (Figures 7A–C). This might be due to underlying neural processes or the different feature space in combination with the regularization strategy or both. In addition, we did not find differences in EEG prediction between tasks (Figure 7D, *p* > 0.11, uncorrected, sign-rank test, two-tailed) but tendencies were similar to decoding results (e.g., Figures 6A,B) likely reflecting the higher sensitivity of decoding analyses that pools information across EEG channels. The distribution of EEG prediction performance across channels was consistent across tasks and encoding models (Figures 7A–C, upper left panels) suggesting similar neural processing sites located in temporal cortex.

## Discussion

In this work, we combined a previously validated ASA behavioral paradigm employing polyphonic music (Disbergen et al., 2018) with EEG-based sound-envelope tracking methods (see, for a comparable approach, Hausfeld et al., 2018) to investigate the contribution of top-down attention mechanisms to ASA. During EEG recordings, participants were presented with polyphonic music and asked to detect a triplet pattern located within or across a bassoon and cello instrument (Figure 1).

Results indicated that the EEG signal tracked the sound envelope of musical instruments. For the segregation tasks, we found that the envelopes of the attended instruments were reconstructed better than those of the unattended ones as has also been reported for a music-in-noise task similar to the present one but with more complex distractors (Greenlaw et al., 2020). These effects were restricted to the delay windows of 150–220 ms, 320–360 ms, and 410–450 ms (Figure 4). Further comparisons for each individual instrument revealed that for our multi-delay models, only the envelopes of the bassoon were reconstructed better when attended to versus unattended (Figure 5A). Results of the time-resolved (i.e., single-delay) analyses showed that both bassoon and cello representations were modulated by the task during a middle-latency window of 160–220 ms for bassoon and 150–210 ms for cello (Figure 5B). Additionally, the bassoon envelope tracking was modulated by task during a late-latency window at 320–380 ms (Figure 5B). While the tracking profile of the bassoon showed overall higher envelope reconstruction, the shape of the profiles was similar with two peaks of high reconstruction at ∼80–100 ms and 160–180 ms. We interpret significant reconstruction at negative delays as reflecting envelope auto-correlations and/or a general temporal attention effect to facilitate processing of the upcoming note (thus reflecting the predictability of the music’s temporal structure). A leave-channel-out analysis indicated the relevance of each EEG channel for envelope tracking, which indicated that temporal channels contributed strongest to the envelope tracking (Figure 5C); topographies were similar for the envelope tracking of the bassoon and cello both when attended to or unattended. For the aggregate task, in contrast to our hypothesis, we did not find any attention effect for aggregate tracking. That is, for both multi-delay and single-delay models, we did not find a difference in the tracking of the aggregate envelope between integration and segregation tasks (Figure 6). An additional EEG prediction analysis showed that, for all tasks, the best fitting model among seven model alternatives was one that included predictors of both the bassoon and cello instrument but not the aggregate (Figure 7).

### Stream Segregation of Instruments and Speakers

Most previous studies employing an EEG-based tracking of sound envelopes examined speech segregation in multi-speaker environments and found that acoustically driven mechanisms dominate effects at delays below approximately 100 ms. For example, those examining temporal response functions (e.g., Crosse et al., 2015), indicated that initial peaks below 100 ms were not modulated by attention, whereas they were by acoustical changes (Ding and Simon, 2012a). In addition, research examining the processing of multiple unattended sounds provided evidence that during delays below 100 ms, unattended sounds remain segregated based on their acoustics, while they get merged based on other factors only during later processing stages (Puvvada and Simon, 2017; Hausfeld et al., 2018). Consistently, the present study found that modulation by attention mainly occurred during later stages of auditory processing. In early processing windows, envelope tracking performance was high but was not modulated by attention. This result is in agreement with the aforementioned speech-based ASA studies and provides a complimentary observation, suggesting similarities between speech and music regarding the early late bisection of attentional selection.

At those time-points during which a significant difference was observed between the attended and unattended envelope tracking of individual instruments, we did not observe changes in the importance of EEG-channels to this envelope tracking when an instrument was attended to or not. The single-delay tracking profiles, both for general as well as instruments specific effects, were very similar between the attended and unattended condition, appearing to be enhanced when sources were attended to. Taken together, these observations suggest that observed effects reflect modulations of a very similar cortical network, which possibly relates to the temporal-frontal network observed in a previous fMRI study (Disbergen, 2020, chapter 3). Their results showed that the listener’s attended instrument could be decoded above chance at the individual subject level from the activity of frontal-temporal auditory networks, comprising large sections of the superior and medial temporal gyrus (STG, MTG), including the HG, planum polare (PP), and planum temporale (PT), sections of the inferior parietal lobe including the angular gyrus (AG), as well as varying portions of the medial and inferior frontal cortex among which the inferior frontal gyrus (IFG). Based on these observations, the attention modulations detected in the present study are potentially located in auditory cortex and arise from signals originating from the medial and inferior frontal cortical regions.

Observations made here concerning the relatively late first occurrence of attention effects suggests that there are contributions of feedback processes at play to the representation and processing of music streams within a multi-instrument environment. One possible interpretation of this points toward a dual-stage contribution of the (early) auditory areas, a first acoustically (i.e., bottom-up) driven feed-forward analysis followed by further top-down feedback modulations from higher-level auditory or frontal areas. Providing sufficient physical differences between sounds, stimulus segregation would represent the initial feed-forward driven analysis, after which attention may interact with these ongoing bottom-up processes in these areas. Results demonstrated here may support the re-entrant activity model of stimulus representation, where active listening modulates feedback interactions between the primary and non-primary areas, driving adaptive neuronal selection (for a review see, Gilbert and Sigman, 2007). On a network-scale, ASA probably involves a task-dependent multi-level analysis of the stimulus with a dynamic interplay between the bottom-up and, among others, attentive mechanisms (for a review see, Sussman, 2017).

### Polyphonic Music Perception

Different theories of polyphonic music perception have been proposed, among which are the divided attention (Gregory, 1990) and the figure-ground model (Sloboda and Edworthy, 1981). The former suggests that music listeners truly divide attentional resources over the different melodic lines, while the latter poses that undivided attention is focused only on single melodic lines and polyphonic perception is achieved by rapidly alternating between melodic streams. A third dominant theory, which may co-exist with the previous, suggests that listeners perform a true integration of the melodies leading to merged perception (Bigand et al., 2000). Prominent bottom-up cues which are employed in the formation of music streams are (instrument) pitch and timbre (Bregman and Pinker, 1978; Wessel, 1979; Cusack and Roberts, 2000; Deutsch, 2013; Marozeau et al., 2013; McAdams, 2013). Musical notes of the same instrument are potentially first grouped based on combinations of these specific bottom-up cues, followed by interactions with top-down mechanisms.

The single-delay envelope tracking profile from the aggregate condition very much resembled that of the bassoon instrument, suggesting a perceptual dominance for this instrument. From a music-theoretical perspective, the lower voice, in our case the cello, tends to be perceptually subordinate (Crawley et al., 2002), which potentially explains such observations. Even though we cannot directly investigate how participants performed the listening tasks, the aggregate tracking results hint at potential perceptual strategies.

During aggregate tracking we did not find an attention effect. In a second step, we determined for each participant the weighting of single instrument tracking profiles from the aggregate task when fitting their aggregate tracking profile (Supplementary Figure 2). While few participants displayed equivalent weighting for each instrument, most participants showed a stronger bassoon versus cello instrument weighting. This fits the perceptual dominance explanation of the aggregate decoding results mentioned above given that a higher contribution of the upper voice would be required to reflect the neural processing of the perceptually more dominant upper voice (i.e., bassoon). However, this observation may also point at different task strategies employed by the participants for performing the aggregate task. In addition, the higher similarity between the aggregate envelopes and bassoon versus cello envelopes might have contributed to the stronger weighting of the upper voice. Thus, it remains unclear whether the participants focused more on the bassoon but also, in an alternating or integrative manner, the cello instrument. Similar paradigms and stimuli controlling for other acoustic features like loudness or timbre could shed more light on cortical processes during multi-instrument music listening.

### Limitations and Considerations

No behavioral differences were observed between segregation and integration tasks, neither here, during fMRI (Disbergen, 2020 chapter 3), or in a psychoacoustical study (Disbergen et al., 2018). This might, however, be related to an insensitivity and/or ceiling effect of the performance metric; please see Disbergen et al. (2018) for a more elaborate discussion on this as well as other task-related considerations. While most results are derived from an EEG tracking analysis (i.e., decoding or backward modeling), we performed an EEG prediction analysis (i.e., encoding or forward modeling) to account for co-variation between instrument predictors. However, encoding analyses do not account for co-variation between EEG channels. Methods like canonical correlation analysis canonical correlation analysis or Regularized Reduced Rank Regression (de Cheveigné et al., 2018; Svanera et al., 2019) that take correlations both at the predictor/feature and channel level into account could provide further insights. Across EEG analyses, we found less tracking performance for models representing envelopes of the lower music voice (i.e., cello), when compared to the upper music voice (i.e., bassoon). Such differences may be related to a general upper-voice dominance effect in the perception of polyphonic music, caused by, for example, its higher pitch (salience) or general loudness effects (Palmer and Holleran, 1994; Fujioka et al., 2005). Perceptually, there may be a continuous loudness difference between voices due to our equalization method based on RMS, as opposed to perceptual matching. In addition, our analysis focused on rapid sound envelope fluctuations which are more pronounced for the bassoon as its envelope slopes are typically steeper than those of cello due to its faster attack and decay times. Even though such factors may contribute to tracking capacity differences between instruments, they do not impact the observed attention effects since these represent task-modulations on the model tracking performance of the same instrument.

In the present study, no attention effect was found for the tracking of the aggregate envelope. Detection of such an effect might be impeded by the specific task performed during the aggregate condition. Assuming that the same neuronal populations represent both instruments during segregation as well as the integration tasks, the difference between segregation and integration tasks may result only in very minor neural differences. During the integration task, neurons could, for example, pool attentional resources more equally across those instrument-specific neuronal populations, which during segregation conditions are up- and/or down-regulated. This may result in small changes which are difficult to detect with EEG in combination with our analysis method. We did observe a within-instrument attention effect, showing that the method is sensitive to attentional changes *per se*, albeit the differences between attending and ignoring sound sources are expected to be larger.

Because the listening tasks did not require continuous attention allocation toward the required instrument, participants may not have paid attention to the instructed instrument(s) during the full stimulus duration. Alternatively, they could have been rapidly alternating attention between the different instruments, especially in the integration task, supported by the observation that for most participants best-fitting encoding models included bassoon and cello predictors but not the aggregate predictor (Figure 7). Based on previous experiments employing this paradigm, we believe that the capacity to detect triplets both within and across voices indicates that participants were capable of segregating and integrating the instruments. Triplet detectability under both conditions provides evidence that they managed to segregate the instruments into their individual streams. In case segregation would not have taken place, they would not have been able to respond correctly whether triplets were present within individual instruments or not. Without segregation, instruments would only differ concerning their tone on and offsets (i.e., rhythmic cues), making it impossible to assign triplets to a single voice. In general, with this paradigm we aimed at investigating which neural mechanisms permit listeners to perceive segregated or integrated melodic voices even though the acoustical signal arriving in their ear consists of the same identical mixed waveform under all conditions (see also Disbergen et al., 2018).

## Conclusion

Employing an envelope tracking method for EEG data, we showed that within a music ASA paradigm the attended music instruments can be significantly better reconstructed than the unattended ones. Attention effects were found during delays indicative of top-down driven modulations onto the ongoing stimulus representations. Effects were shown both when testing a generalized attention effect across instruments as well as for all the individual instruments. No attention effect was found for aggregate tracking, even though two distinct subgroups of participants emerged when fitting the aggregate single-delay tracking profile by a linear combination of the instrument tracking profiles. Our results extend the attentive modulation of speech envelopes in ASA into the domain of music stimuli. Furthermore, these findings suggest that similar effects previously observed with fMRI are potentially driven by top-down modulations, possibly modulating the later processing in (early) auditory cortical areas. Further research with MEG or ECoG promises sufficient localization of neural effects while preserving the temporal precision needed to shed further light onto the underlying neuronal processes of those effects which were observed both here with EEG and, previously, with fMRI.

## Data Availability Statement

The data supporting the conclusions of this article are available at DataVerseNL (https://doi.org/10.34894/9ITCNN).

## Ethics Statement

The studies involving human participants were reviewed and approved by the Ethics Review Committee Psychology and Neuroscience (#167_09_05_2016), Faculty of Psychology and Neuroscience, Maastricht University. The participants provided their written informed consent to participate in this study.

## Author Contributions

LH, ND, and RZ contributed to the conception and design of the study. ND, RZ, EF, and GV provided the stimuli and learning paradigm. LH performed the data acquisition and processing. LH and ND performed the statistical analyses and wrote the first draft of the manuscript. LH, ND, GV, RZ, and EF contributed to manuscript revision. All authors contributed to the article and approved the submitted version.

## Conflict of Interest

The authors declare that the research was conducted in the absence of any commercial or financial relationships that could be construed as a potential conflict of interest.

## Publisher’s Note

All claims expressed in this article are solely those of the authors and do not necessarily represent those of their affiliated organizations, or those of the publisher, the editors and the reviewers. Any product that may be evaluated in this article, or claim that may be made by its manufacturer, is not guaranteed or endorsed by the publisher.

## Supplementary Material

Supplementary Figure 1 Example stimuli and decoding predictors. **(A)** Isolated quarter and eighth notes typical of the note range used in the compositions (bassoon A_4_, cello E_3_); notes are synthesized with the same settings as the compositions. Dotted and solid lines show the envelope and its rectified derivative used in the analyses, background light-colored areas denote the sounds’ waveforms for bassoon (blue) and green (cello). Black lines denote the time interval that a note was “on” according to the midi information. **(B)** 10-s example waveforms (light colored area) and their envelope derivative of bassoon and cello. **(C)** Lines denote the autocorrelation of bassoon, cello and the aggregate predictors (i.e., rectified envelope derivatives) extracted from the waveforms. These reflect the computation of the envelope derivative, which is slowly changing introducing a high correlation between neighboring samples (low-pass filter) for all waveforms. In addition, a high autocorrelation at ±0.5 and ±1 s is observed, which is due to the design of the stimuli (duration/onsets of eighth and quarter notes at 60 bpm).

Supplementary Figure 2 Aggregate tracking results. Fitting the single-delay aggregate tracking profile (a) during the aggregate task from a linear combination of the bassoon and cello instrument tracking profile during this task. Symbols denote the beta coefficients of the linear fit for the bassoon (*x*-axis) and cello instrument (*y*-axis) for each participant. Applying a criterion (bassoon-cello coefficient > 0.01) displays that the statistically higher bassoon versus cello instrument weighting across participants (see text) can be found in most individuals (G2, squares, *N* = 10).

Supplementary Table 1 Overview behavioral results during testing session. This table shows the participants’ performances for both the integration and segregation tasks (i.e., aggregate task and bassoon/cello tasks, respectively). In addition to accuracy, false alarm (FA) rates and their absolute differences are presented (“FA difference”). Both accuracy and FA differences were used to identify outliers (see section “Participants” and Figure 3). The latter were used for potential response biases indicative of undesired strategies during task performance. Black and gray fonts denote included and excluded participants, respectively. Red font indicates values important for exclusion decision. “AVG 19” and “AVG 15” indicate average performance values with all participants and five participants excluded, respectively.
