# Supplementary figures and images for: Modulating Cortical Instrument Representations During Auditory Stream Segregation and Integration With Polyphonic Music
Source: Front Neurosci. 2021 Sep 24;15:635937. doi: 10.3389/fnins.2021.635937 (PMC8498193; doi:10.3389/fnins.2021.635937)

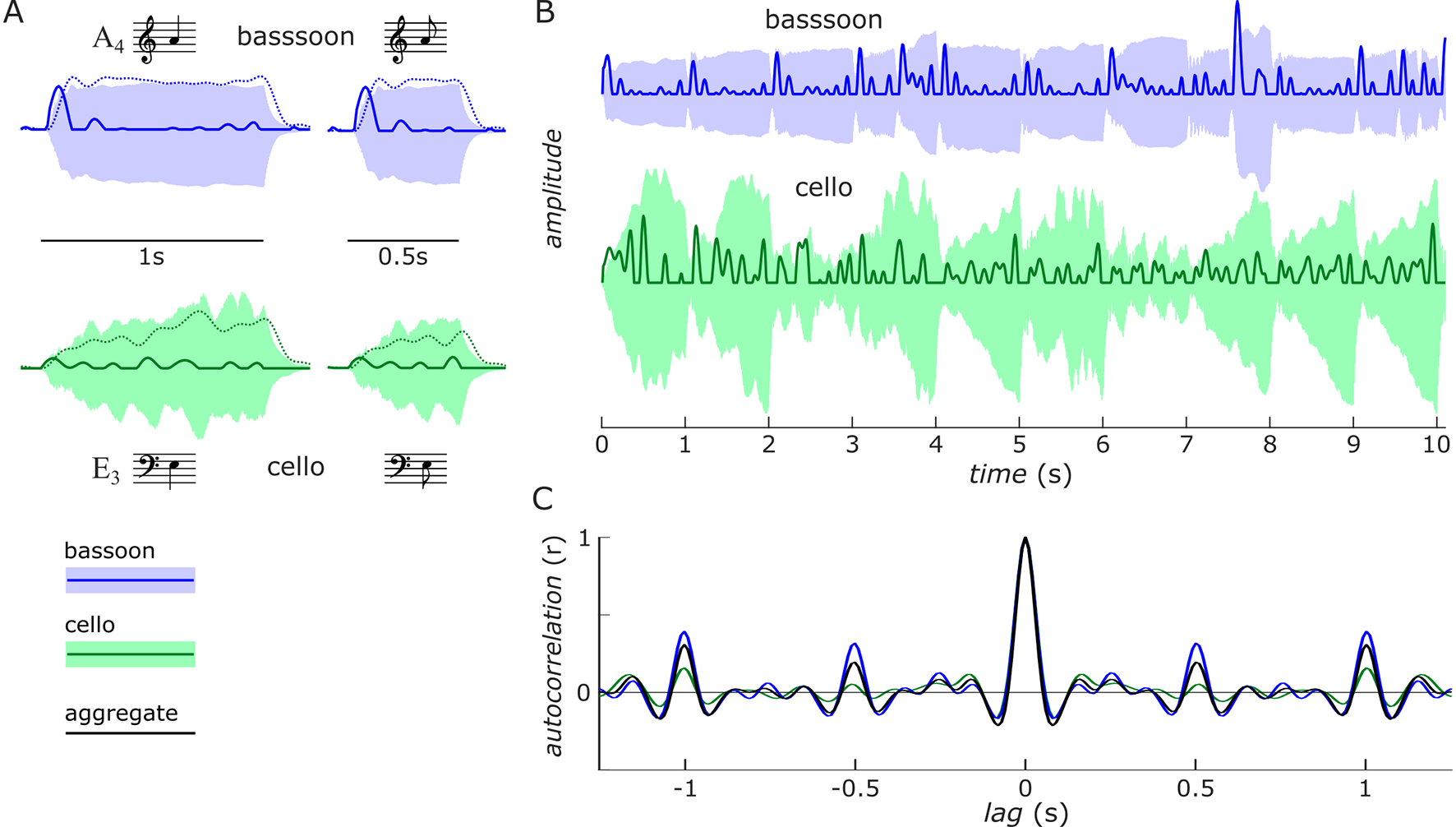

Supplement: Supplementary Figure 1 — Example stimuli and decoding predictors. (A) Isolated quarter and eighth notes typical of the note range used in the compositions (bassoon A4, cello E3); notes are synthesized with the same settings as the compositions. Dotted and solid lines show the envelope and its rectified derivative used in the analyses, background light-colored areas denote the sounds’ waveforms for bassoon (blue) and green (cello). Black lines denote the time interval that a note was “on” according to the midi information. (B) 10-s example waveforms (light colored area) and their envelope derivative of bassoon and cello. (C) Lines denote the autocorrelation of bassoon, cello and the aggregate predictors (i.e., rectified envelope derivatives) extracted from the waveforms. These reflect the computation of the envelope derivative, which is slowly changing introducing a high correlation between neighboring samples (low-pass filter) for all waveforms. In addition, a high autocorrelation at ±0.5 and ±1 s is observed, which is due to the design of the stimuli (duration/onsets of eighth and quarter notes at 60 bpm). [file Image_1.TIFF]

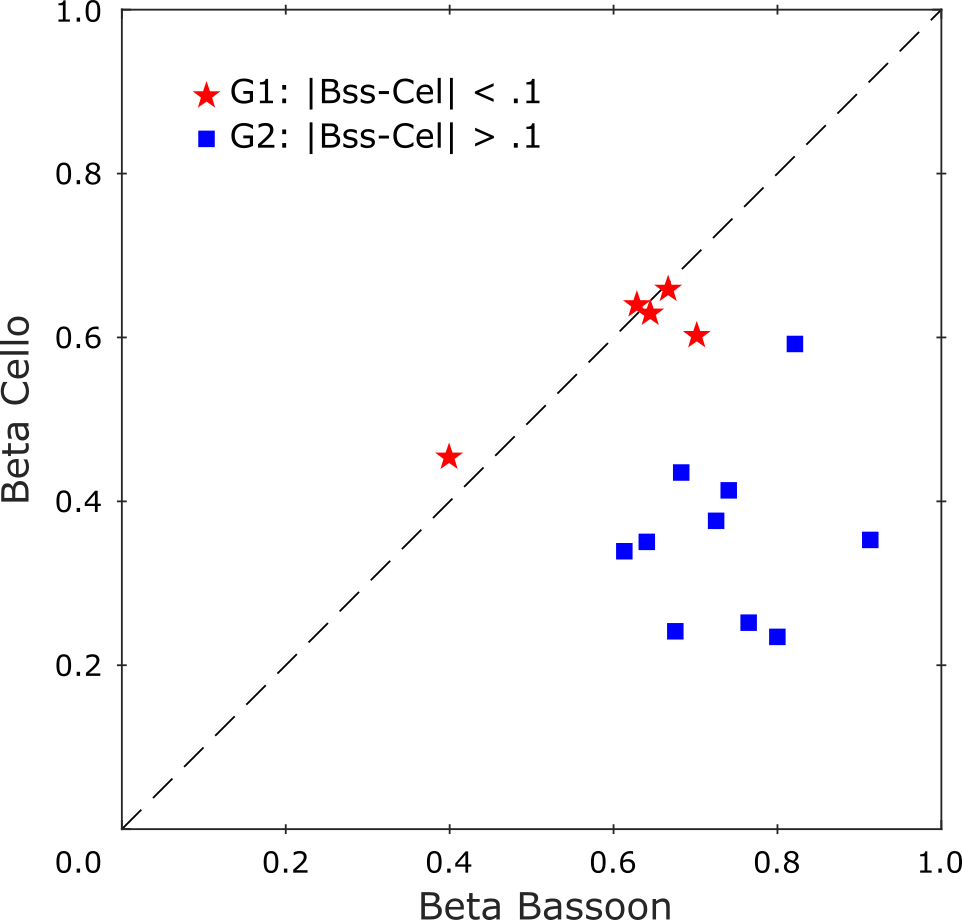

Supplement: Supplementary Figure 2 — Aggregate tracking results. Fitting the single-delay aggregate tracking profile (a) during the aggregate task from a linear combination of the bassoon and cello instrument tracking profile during this task. Symbols denote the beta coefficients of the linear fit for the bassoon (x-axis) and cello instrument (y-axis) for each participant. Applying a criterion (bassoon-cello coefficient > 0.01) displays that the statistically higher bassoon versus cello instrument weighting across participants (see text) can be found in most individuals (G2, squares, N = 10). [file Image_2.TIFF]
