# Supplementary material for: Modulating Cortical Instrument Representations During Auditory Stream Segregation and Integration With Polyphonic Music
Source: Front Neurosci. 2021 Sep 24;15:635937. doi: 10.3389/fnins.2021.635937 (PMC8498193; doi:10.3389/fnins.2021.635937)
Supplement: Supplementary Table 1 — Overview behavioral results during testing session. This table shows the participants’ performances for both the integration and segregation tasks (i.e., aggregate task and bassoon/cello tasks, respectively). In addition to accuracy, false alarm (FA) rates and their absolute differences are presented (“FA difference”). Both accuracy and FA differences were used to identify outliers (see section “Participants” and Figure 3). The latter were used for potential response biases indicative of undesired strategies during task performance. Black and gray fonts denote included and excluded participants, respectively. Red font indicates values important for exclusion decision. “AVG 19” and “AVG 15” indicate average performance values with all participants and five participants excluded, respectively. [file Table_1.pdf]

	ACCURACY				FALSE ALARM RATE					
	task				bassoon task			cello task		
**participant**	aggregate	bassoon	cello	*AVERAGE*	cello triplet	no triplet	FA difference	bassoon triplet	no triplet	FA difference
S1	1.000	0.850	0.925	*0.925*	0.200	0.200	0.000	0.000	0.000	0.000
S2	0.950	0.950	0.925	*0.942*	0.000	0.182	0.182	0.083	0.125	0.042
S3	0.850	0.700	0.700	*0.750*	0.200	0.100	0.100	0.300	0.300	0.000
S4	0.900	0.667	0.600	*0.722*	0.556	0.500	0.056	0.500	0.286	0.214
S5	0.950	0.875	0.950	*0.925*	0.100	0.000	0.100	0.100	0.100	0.000
S6	0.850	0.700	0.850	*0.800*	0.333	0.636	0.303	0.250	0.375	0.125
S7	0.900	0.900	0.925	*0.908*	0.091	0.111	0.020	0.000	0.091	0.091
S8	0.975	1.000	1.000	*0.992*	0.000	0.000	0.000	0.000	0.000	0.000
S9	0.850	0.925	0.975	*0.917*	0.182	0.111	0.071	0.000	0.091	0.091
S10	0.875	0.800	0.900	*0.858*	0.444	0.273	0.172	0.182	0.222	0.040
S11	0.800	0.725	0.775	*0.767*	0.600	0.500	0.100	0.400	0.500	0.100
S12	0.775	0.750	0.825	*0.783*	0.636	0.333	0.303	0.182	0.444	0.263
S13	1.000	0.975	1.000	*0.992*	0.000	0.000	0.000	0.000	0.000	0.000
S14	0.950	0.925	0.950	*0.942*	0.000	0.200	0.200	0.000	0.100	0.100
S15	0.775	0.825	0.825	*0.808*	0.111	0.000	0.111	0.273	0.111	0.162
S16	0.825	0.825	0.875	*0.842*	0.273	0.333	0.061	0.111	0.273	0.162
S17	0.775	0.775	0.800	*0.783*	0.200	0.300	0.100	0.200	0.300	0.100
S18	0.900	0.500	0.625	*0.675*	0.875	0.833	0.042	0.545	0.778	0.232
S19	1.000	1.000	1.000	*1.000*	0.000	0.000	0.000	0.000	0.000	0.000
AVG 19	0.889	0.825	0.864	*0.860*	0.253	0.243	0.101	0.165	0.216	0.091
AVG 15	0.898	0.870	0.902	*0.890*	0.160	0.133	0.081	0.089	0.122	0.059

Supplementary Table 1. Overview Behavioral Results During Testing Session. This table shows the participants' performances for both the integration and segregation tasks (*i.e.*, aggregate task and bassoon/cello tasks respectively). In addition to accuracy, false alarm (FA) rates and their absolute differences are presented ('FA difference'). Both accuracy and FA differences were used to identify outliers (see Methods - Participants and Fig. 3). The latter were used for potential response biases indicative of undesired strategies during task performance. Red font indicates values important for exclusion decision. Black and grey fonts denote included and excluded participants, respectively. 'AVG 19' and 'AVG 15' indicate average performance values with all participants and five participants excluded, respectively.
